# Evaluation of Acanthamoeba keratitis cases in a tertiary medical care centre over 21 years

**DOI:** 10.1038/s41598-020-80222-3

**Published:** 2021-01-13

**Authors:** Wolfgang List, Wilfried Glatz, Regina Riedl, Georg Mossboeck, Gernot Steinwender, Andreas Wedrich

**Affiliations:** 1 Department of Ophthalmology, Medical University of Graz, 8036 Graz, Austria; 2 Institute for Medical Informatics, Statistics and Documentation, Medical University of Graz, 8036 Graz, Austria

**Keywords:** Eye diseases, Medical research

## Abstract

To report on Acanthamoeba keratitis cases in a tertiary university eye-hospital in Graz, Austria, over a 21-year period. Retrospective study. Parameters included demographics, diagnostics, clinical courses, medical therapies, surgical interventions, secondary complications, and best spectacle-corrected visual acuity (BSCVA). Patient records for 44 eyes of 42 patients were analysed; 2 bilateral infections. Mean age at presentation was 31 ± 13 (16–65) years; contact lenses were used in 41 of 44 eyes (93.2%). Symptoms at initial presentation were mainly pain (41/43, 95.3%) and photophobia (16/43, 37.2%). Most frequent morphological findings were stromal infiltrates (30/44, 68.2%). Diagnosis was mainly confirmed by smears (40/42, 95.2%) and polymerase chain reaction (8/42, 19%). Antiamoebic treatment comprised biguanides and diamidines. Penetrating keratoplasty was performed in 10/44 (22.7%) eyes. Median time from symptom onset to initial visit was 2 (0–26) weeks; median follow-up was 30 (2–1008) weeks. BSCVA improved in 23/36 (63.9%) eyes, remained unchanged in 6/36 (16.7%) eyes and deteriorated in 7/36 (19.4%) eyes. Acanthamoeba keratitis predominantly occurs in young contact lens wearers. Diagnosis should be considered in patients with pain and stromal infiltrates. In the majority of cases, BSCVA can be improved. Early diagnosis and adequate treatment should be implemented to prevent complications.

## Introduction

Acanthamoeba keratitis (AK) is a rare disease accounting for only 2% of corneal infections. However, the incidence has been consistently increasing^1–5^. The use of contact lenses (CLs) and corneal trauma leading to a loss of integrity of the epithelium are the leading risk factors^6–12^. Acanthamoeba are ubiquitous; they can be present in running water and bottled water, swimming pools, air conditioning systems, and even soil^13–16^.

Due to nonspecific clinical presentations, similarities to other corneal pathologies, especially herpes simplex virus (HSV) keratitis, and the necessity of complex diagnostic testing, an accurate diagnosis is often challenging and therefore delayed^12^. Thus, AK might be associated with long-term complications, such as persistent corneal defects causing reduced vision, potentially leading to blindness^17,18^. Occasionally, surgical interventions, including penetrating keratoplasty (PKP), are necessary to restore good visual acuity, and in rare cases enucleation may be the only option to relieve pain^19^.

In this retrospective study, we evaluated cases of AK at the Department of Ophthalmology at the Medical University of Graz, Austria, focusing on demographics, diagnostics, clinical courses, medical therapies, surgical interventions, and secondary complications.

## Results

### Demographics

Forty-four eyes of 42 patients with initial presentation between 1997 and 2018 were included in this retrospective study. Two patients were diagnosed with bilateral infections. During the study period, a maximum annual number of 5 cases was observed between the years 2011 and 2015. An analysis of monthly AK cases showed cases peaking in summer, especially in August, with n = 9 cases (including 1 bilateral infection).

The patient characteristics are presented in Table 1. The mean age at first presentation was 31 ± 13 years, ranging from 16 to 65 years. In 41 of the cases (93.2%), CLs were used (25 soft CLs, 8 rigid CLs and 8 cases with an unknown CL type). In two cases, a CL-associated history of trauma was reported. Another case developed AK after LASIK treatment. In four cases a recent history of bathing or swimming in pools, lakes or seas was given, and in one case regular flushing of the CL container with tap water was noted.

**Table 1:** Patient characteristics.

	N total	N mean ± SD	% med (min–max)
**Characteristics**			
Age, years	44	31 ± 13	27 (16–65)
Female	44	20	45.5
Eye, OD	44	23	52.3
Bilateral AK	44	4	9.1
CLs	44	41	93.2
**Self-reported symptoms**			
Pain	43	41	95.4
Photophobia	43	16	37.2
Foreign body sensation	43	10	23.3
Epiphora	43	8	18.6
Blepharospasm	43	2	4.7
**Morphological pathology**			
Stromal infiltration	44	30	68.2
Anterior synechia	44	3	6.8
Hypopyon	44	2	4.6
Advanced AK signs^a^	44	30	68.2
**Diagnosis-methods**			
Smear	42	40	95.2
PCR	42	8	19.1
AC puncture	44	2	4.5
Biopsy	41	1	2.4
**Microbial results**			
Cyst	31	24	77.4
Bacterial coinfection	30	19	63.3
Fungal coinfection	29	3	10.3
HSV	32	3	9.4

^a^Defined as ≧ 1 of the following signs presented: ring infiltrate, stromal impairment, hypopyon; *AC* anterior chamber, *AK* acanthamoeba keratitis, *CL* contact lens, *max* maximum, *med* median, *min* minimum, *N* absolute number, *PCR* polymerase chain reaction, *SD* standard deviation.

### Clinical presentation and diagnosis

The clinical presentations and diagnoses are summarized in Table 1. Self-reported symptoms at initial presentation were documented in 43 cases and included pain (41/43, 95.3%), photophobia (16/43, 37.2%), foreign body sensation (10/43, 23.3%), epiphora (8/43, 18.6%), and blepharospasm (1/43, 4.7%). The most frequent morphological finding was stromal infiltration, including ring infiltrates (30/44, 68.2%). Anterior synechiae and hypopyons were observed in 6.8% and 4.6% of the cases, respectively. In 30/44 (68.2%) eyes, at least one physical sign of advanced AK was observed.

**Figure 1:**
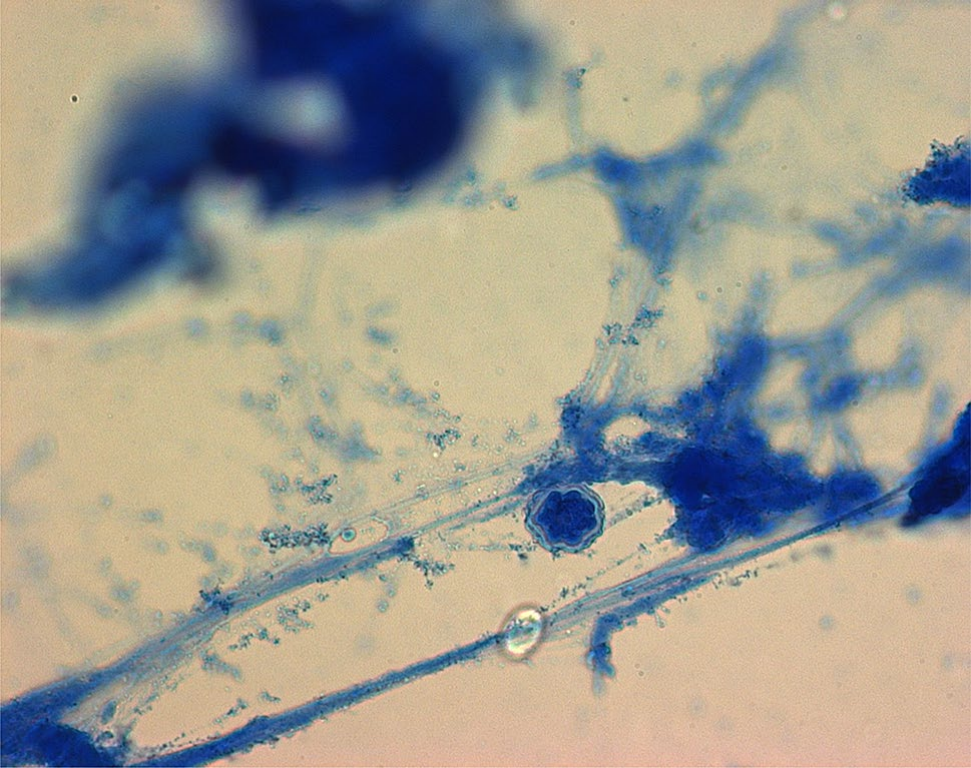
Acanthamoeba cyst.

The diagnosis of AK was mainly confirmed by smears (40/42, 95.2%), followed by PCR (8/42, 19%). Anterior chamber puncture (2/44, 4.5%) and biopsies (1/41, 2.4%) played subordinate roles. In 34/42 (81%) eyes, only smears were performed for AK testing. Acanthamoeba cysts were observed in 24/31 (77.4%) eyes (Fig. 1). In 32/44 (72.7%) cases, coinfections were observed, including 19/30 (63.3%) bacterial, 3/29 (10.3%) fungal, and 3/32 (9.4%) HSV infections. Bacterial culture testing was performed in 13/19 (68.4%) cases. The results included *Staphylococcus haemolyticus*, *Bacillus cereus*, *Staphylococcus epidermidis*, *Propionibacterium acnes*, *Staphylococcus capitis*, *Enterobacter cloacae*, *Stenotrophomonas maltophilia*, *Staphylococcus aureus*, *Bacillus simplex* and *Staphylococcus xylosus*.

### Treatment

AK treatment involves AAT with concomitant ointments. The majority of patients were treated with a combination of biguanides and diamidines (32/44, 72.7%); however, monotherapy (11/44, 25%) with biguanides was documented in 3/44 (6.8%) eyes, and monotherapy with diamidines was documented in 8/44 (18.2%) eyes. In one confirmed case, AK was resolved by topical therapy with antibiotic ointment, antiviral ointment and disinfection (Betaisodona 1:32) without specific AAT. Upon confirmation of the AK diagnosis, therapy with biguanide 0.02% was initiated for all but two eyes (42/44, 95.5%), with a frequency of hourly or shorter in 30 eyes (30/42, 71.4%); meanwhile, therapy with diamidine 0.1% was initiated for all but four (40/44, 90.9%) eyes with a frequency of hourly or shorter in 28 eyes (28/40, 70%). At this point, antibiotic ointments were instilled in 36 eyes (36/44, 81.8%). Topical corticosteroids were administered when the diagnosis of AK was proven in 14 eyes (14/44, 31.8%) and were prescribed within 1 week in 19 eyes (19/44, 43.2%) and within 1 month in 25 eyes (25/44, 56.8%).

Table 2 outlines the different AAT combinations and their frequencies for concomitant treatment, number of appointments and follow-up times. Prior to initial presentation, topical antimicrobial treatment was initiated in 36/44 (81.8%) eyes by external registered ophthalmologists. The median follow-up time was 30 (2–1008) weeks. During follow-up, the median number of appointments per eye was 12.5 (2–70).

**Table 2:** Concomitant medications and follow-up times.

	Monotherapy^a^ N = 11		Only biguanides N = 3		Only diamidines N = 8		Dual therapy^b^ N = 32	
	N mean ± SD	% med (min–max)	N mean ± SD	% med (min–max)	N mean ± SD	% med (min–max)	N mean ± SD	% med (min–max)
Antibiotic therapy	10	90.9	2	66.7	8	100	31	96.9
Topical steroids	7	63.6	1	33.3	6	75	26	81.3
Antiviral therapy	3	27.3	–	–	3	37.5	7	21.9
Antimycotic therapy	–	–	–	–	–	–	6	18.8
Disinfectant eye drops	3	27.3	1	33.3	2	25	5	15.6
Number of appointments	17 ± 18	12 (5–70)	12 ± 5	15 (7–15)	18 ± 21	12 (5–70)	17 ± 12	13.5 (2–45)
Follow-up time, weeks	186 ± 308	29 (2–1008)	88 ± 125	30 (3–231)	223 ± 354	24 (2–1008)	89 ± 132	31 (2–603)

^a^Defined as a monotherapy with either biguanides or diamidines.

^b^Defined as the combination of biguanides and diamidines in the same eye during the course of therapy.

*max* maximum, *med* median, *min* minimum, *N* absolute number, *SD* standard deviation.

### Visual acuity and IOP

**Figure 2:**
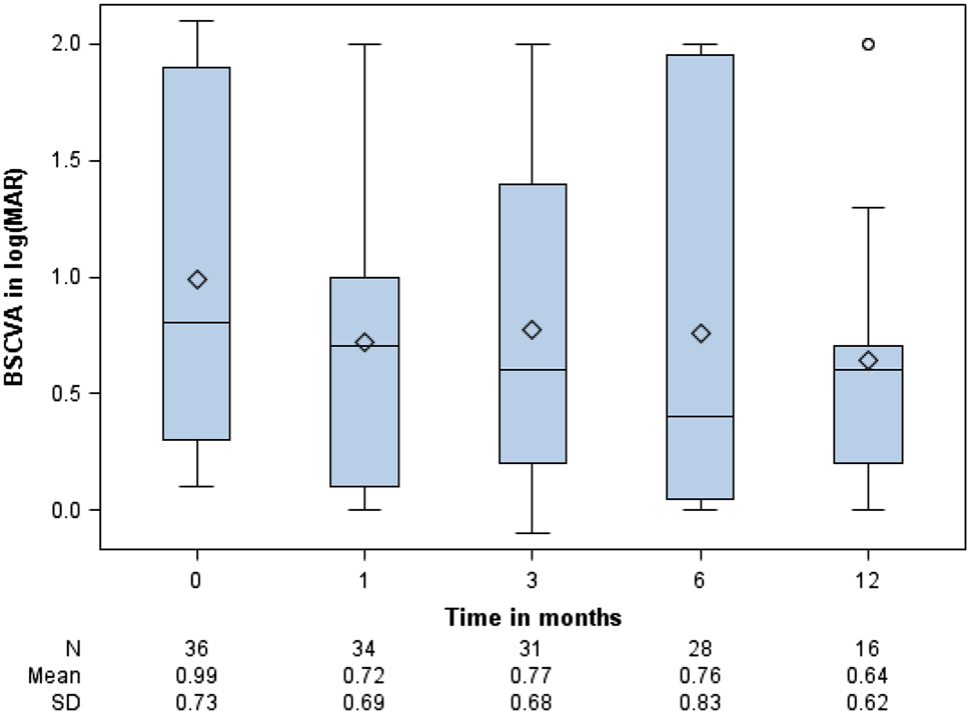
Boxplots of best spectacle-corrected visual acuity (BSCVA) courses within the first 12 months measured at initial presentation, after 1 month, after 3 months, after 6 months and after 12 months. The table in the bottom indicates the absolute number (N) of available data, mean value and standard deviation.

The mean BSCVA at first presentation was log(MAR) 0.99 ± 0.73 (n = 36), and it improved to log(MAR) 0.72 ± 0.69 (n = 34) after 1 month. After 1 year, a BSCVA of log(MAR) 0.64 ± 0.62 (n = 16) was observed (Fig. 2). The final BSCVA was log(MAR) 0.56 ± 0.72 (n = 44). Among 36 eyes with documented initial BSCVA, the final BSCVA improved in 23 (63.9%) eyes, remained unchanged in 6 (16.7%) eyes and deteriorated in 7 (19.4%) eyes.

The improvement from initial to final BSCVA was significant (0.39 ± 0.68, *p* = 0.001). In 5/44 (11.4%) eyes, the final BSCVA was hand movement or worse.

Initial IOP was measured in only 9 eyes, resulting in a mean of 19.2 ± 6.2 mmHg. After 12 months, a mean IOP of 13.4 ± 4.6 mmHg was observed in 8 eyes.

### Onset time of symptoms to initial visit

The median time from symptom onset to the initial visit was 2 (0–26, n = 40) weeks. Significant differences in the time delay were observed between males with 4 (0–26) weeks and females with 1 (0–13) week (*p* = 0.012). Further tendencies were observed between the presence or absence of cysts, with a median time of 1 (0–13) week if present and 4 (0–26) weeks if absent (Table 3).

**Table 3:** Time from symptom onset to initial visit.

	N	Mean ± SD	Med (min–max)	*p* value
**Age**^**a**^				
≦ 27	19	2.2 ± 2.2	1 (0–8)	0.209
> 27	21	4.6 ± 6.0	3 (0–26)	
**Sex**				
Male	21	4.5 ± 5.4	4 (0–26)	0.012
Female	19	2.3 ± 3.6	1 (0–13)	
**Cysts**				
No	6	7.9 ± 9.6	4 (0–26)	0.087
Yes	23	2.6 ± 3.3	1 (0–13)	
**Advanced AK**				
No	14	3.7 ± 6.6	1 (0–26)	0.577
Yes	26	3.3 ± 3.4	2 (0–13)	

^a^Categorization is based on a median age of 27 among all cases.

*AK *Acanthamoeba keratitis, *max* maximum, *med* median, *min* minimum, *N* absolute number, *SD* standard deviation.

### Surgical interventions, outcomes and complications

In 34 (77.3%) cases, infections were treated by topical treatment alone, whereas 10 (22.7%) eyes underwent PKPs. In three cases, PKP had to be repeated. The median time from initial presentation until the first PKP was 44 (5–928) weeks.

The necessity for PKP was associated with the absence of cysts (*p* = 0.029) and a long time from symptom onset to the initial visit (*p* = 0.004) (Table 4).

**Table 4:** Frequency of penetrating keratoplasties.

	No PKP N = 34 (77.3%)		PKP N = 10 (22.7%)		*p* value
	N mean ± SD	% med (min–max)	N mean ± SD	% med (min–max)	
Age	29 ± 11	26 (16–61)	37 ± 16	34 (18–65)	0.079
Female	16	47.1	4	40.0	0.734
Cysts	21	87.5	3	42.9	0.029
Advanced AK	22	64.7	8	80.0	0.462
Time from symptom onset to initial visit, weeks	2.2 ± 2.2	2 (0–8)	8.3 ± 8.2	4 (1–26)	0.004

*AK* Acanthamoeba keratitis, *max* maximum, *med* median, *min* minimum, *N* absolute number, *PKP* penetrating keratoplasty, *SD* standard deviation.

**Figure 3:**
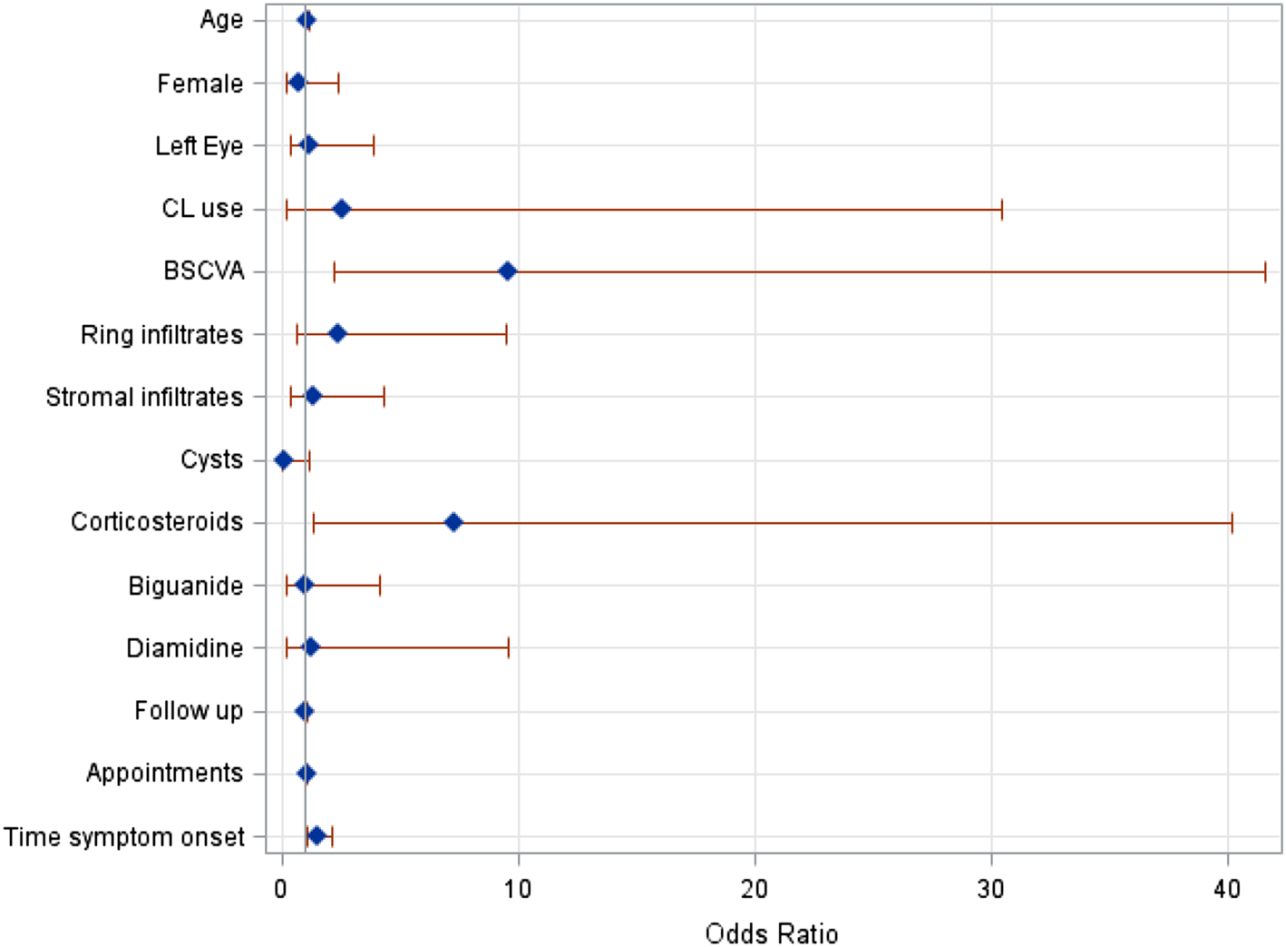
OR for the risk factors for poor outcome.

Poor outcomes were found in 24/44 (54.5%) eyes, and the results are summarized in Table 5. In our analysis, poor outcomes were associated with corticosteroid use (91.7% vs. 60%, odds ratio [OR]: 7.33), older age (34.6 ± 15.0 vs. 26.2 ± 8.2 years, OR: 1.06), poor BSCVA at initial presentation (log(MAR) 1.35 ± 0.69 vs. log(MAR) 0.49 ± 0.42, OR: 9.59), an increased follow-up time (84, 2–2008 vs. 17, 2–174 weeks, OR: 1.02) and the duration of symptoms (4, 0–26 vs. 1, 0–8 weeks, OR: 1.50); however, no association was found with the number of appointments (21 ± 17 vs. 14 ± 8, OR: 1.05). In Fig. 3, the ORs for these risk factors are presented graphically.

**Table 5:** Univariable logistic regression analyses for ‘poor outcome’.

	Poor outcome N = 24		Good treatment response N = 20		OR	95% CI
	N mean ± SD	% med (min–max)	N mean ± SD	% med (min–max)		
Age	34.6 ± 15.0	29.5 (16–65)	26.2 ± 8.2	24.5 (16–45)	1.06	(1.00, 1.13)
Female	10	41.7	10	50.0	0.71	(0.22, 2.36)
Left Eye	13	54.2	10	50.0	1.18	(0.36, 3.88)
CL use	23	95.8	18	90.0	2.56	(0.21, 30.47)
BSCVA at initial visit, log(MAR)	1.35 ± 0.69	1.6 (0.1–2.1)	0.49 ± 0.42	0.3 (0.1–1.5)	9.59	(2.21, 41.58)
Hypopyon	2	8.3	0	0.0	–	–
Ring infiltrates	9	37.5	4	20.0	2.40	(0.61, 9.46)
Stromal infiltrates	13	56.5	10	50.0	1.30	(0.39, 4.33)
Cysts	10	62.5	14	93.3	0.12	(0.01, 1.15)
Corticosteroids	22	91.7	12	60.0	7.33	(1.34, 40.21)
Biguanides	19	79.2	16	80.0	0.95	(0.22, 4.15)
Diamidines	22	91.7	18	90.0	1.22	(0.16, 9.56)
Follow-up, weeks	209.3 ± 255.4	84.1 (1.6–1008.3)	27.6 ± 40.1	17.2 (1.6–174.4)	1.02	(1.00, 1.03)
Appointments	20.7 ± 16.9	14 (4–70)	13.7 ± 7.8	12 (2–30)	1.05	(0.99, 1.11)
Time from symptom onset to initial visit, weeks	5.0 ± 5.7	4.0 (0.1–26.0)	1.6 ± 2.1	0.7 (0.0–8.0)	1.50	(1.05, 2.16)

*BSCVA* best spectacle-corrected visual acuity, *CL* contact lens, log(MAR): logarithm of the minimum angle of resolution, *max* maximum, *med* median, *min* minimum, *N* absolute number, *SD* standard deviation, *OR* odds ratio, *CI* confidence interval.

Secondary glaucoma requiring treatment was found in 4 (9.1%) eyes, and significant cataracts were found in 7 (15.9%) eyes.

## Discussion

AK is a rare corneal parasitic disease that is potentially sight threatening and mainly affects young CL wearers. These results are in accordance with those of previous studies^4,20–23^.

The aim of this study was to analyse AK cases in our department between 1997 and 2018 and compare the characteristics, treatment strategies, results and outcomes with other studies.

An increase in AK incidence was discussed in several international studies^20,24–26^. In our study population, we observed a peak in first presentations during the summer months, especially the month of August. We suppose that CL use, especially the use of disposable CLs, is higher and that leisure time activities are more common in summer than in other seasons. This is in accordance with multiple studies that found AK to be most frequent in summer^27–29^. Other reasons that have been discussed are increases in Acanthamoeba presence in surface water and domestic water during summer, which might especially affect CL wearers in the UK who use domestic rooftop cisterns, which may have an extraordinarily high population of Acanthamoeba^28,30^. Another possible explanation might be the increase in excystation into trophozoites, which is optimized at approximately 30 degrees Celsius in vitro^31^. In contrast, a study from Manchester reported an increase in winter, but they assumed that travelling to warmer climates for vacation might have been the reason for their results^32^. We therefore maintain the hypotheses that AK is associated with CL wear and increased exposure to water, swimming pools and leisure time activities. In our study population, we observed an exposure to water in association with bathing in four cases; additionally, in one case frequent flushing of the CL container with tap water was described. As these numbers appear to be small, we need to consider the retrospective study design and assume the influence of water to be higher.

We found that CLs used to be a major risk factor for AK, as 93.2% of patients were CL wearers; nearly similar results were reported in two 7-year reviews of AK in Auckland^4,20^. Among the CLs, 61% were soft CLs and 19.5% were hard CLs. Interestingly, one of the two bilateral cases had no association with CL use, and the origin of the bilateral infection remains unclear. These findings also contradicted the results of McKelvie et al*.*^20^ who reported 100% CL use in bilateral cases. In our population, the bilateral non-CL-associated case was previously hospitalized in another ophthalmic centre in Austria, where corneal smears were performed for both eyes. Although trauma was negated, corneal scraping might also be considered corneal trauma. Moreover, refractive surgery plays a role in AK; one case occurred after LASIK, and the patient also wore a therapeutic CL.

In a study by Ross et al*.*^23^ a bimodal age distribution was described, with peaks at 16–25 and 56–65 years. In our study population, we had clear AK dominance between 16 and 30 years and a slight peak between 36–45 and 61–65 years. Correspondingly, the peaks and curvatures from our graph and the population by Ross et al*.*^23^ resemble each other quite closely, and they are also similar in terms of CL use (93.2% and 93.3%, respectively).

The first line and gold standard laboratory test for keratitis in our department remains corneal scraping, which was performed in 90.9% of cases. The second most common modality was PCR in 18.2% of the population; PCR has become increasingly popular in recent years. Confocal microscopy is a relatively new diagnostic modality, and it was not being used during the whole study period. Although in vivo confocal microscopy (IVCM) showed superiority in terms of sensitivity and specificity compared with PCR and corneal scraping, it remains a highly user-dependent modality^33^. Hau et al*.*^34^ described a twofold difference in sensitivity between their most experienced and least experienced observers. We therefore adhere to the opinion that IVCM can be a useful non-invasive diagnostic tool but should not displace laboratory tissue testing.

To date, only a few randomized, controlled clinical studies on the treatment of AK have been conducted. Therapeutic strategies are mainly discussed in case series and reports; however, no standardized therapeutic guideline has been agreed upon. In a comparison of polyhexamethylene biguanide and chlorhexidine as monotherapy agents, no differences in therapeutic efficacy and visual acuity (VA) could be found^35^. However, combination therapy involving biguanides, diamidine derivatives and antibiotic ointments is recommended^36,37^. In our cases, the vast majority (32/44, 72.7%) of patients received combined diamidines and biguanides during the course of the disease, whereas monotherapy (11/44, 25%) involving biguanides was applied in 3/44 (6.8%) eyes and monotherapy involving diamidines was applied in 8/44 (18.2%) eyes. At the time when the AK diagnosis was proven and AAT was initiated, the frequency of ointment application was hourly or shorter, or up to once every ten minutes for diamidines and biguanides in the majority of cases. Despite these findings, great variations in treatment strategies were observed in our study population. While AAT was stopped within two weeks of treatment for some patients, other patients were maintained on diamidine 0.1% once a day for over a year to prevent recrudescence. Similar approaches were observed for the prescription of topical corticosteroids.

Concomitant topical therapy was used in all of the treatment groups, with almost no difference in the frequency of antibiotic ointments administered in monotherapy and dual therapy. In contrast, slight differences were found in the frequency of use of topical steroids (63.6% vs. 81.3%) and further disinfectant (Betaisodona) ointments (27.3% vs. 15.6%).

Antimycotic treatment was used in only the dual therapy group, indicating that severe cases might have received dual therapy as the antiamoebic efficacy of topical and systemic antimycotic drugs has been previously demonstrated in vitro and in vivo^38–41^.

We also investigated the associations of patients and AK characteristics with time between onset of symptoms and initial presentation (Table 3). In accordance with previous results, the diagnostic delay was significantly longer for men than for women (*p* = 0.012)^42^. Another tendency was the presence of cysts and a short diagnostic delay. This finding is interesting because cysts are metabolically inactive. None of the remaining characteristics seemed to have an influence on diagnostic delay, even not the presence of advanced AK.

In our population, patients with delayed initial presentation were more likely to undergo PKP than those without delayed initial presentation (*p* = 0.004). Interestingly, the surgically treated patients did not present with advanced stages of AK significantly more than the nonsurgically treated patients (*p* = 0.462). We also observed that the patients with cysts were less likely to undergo PKP (*p* = 0.029) than the patients without cysts. This can be explained by the fact that encysted Acanthamoeba are difficult to treat and are metabolically inactive^43^.

The cut-off criteria for poor outcomes were defined as the final BSCVA equal to or higher than logMAR 0.4, thus less than 0.5, the threshold for obtaining a driving license in Austria^44^ and the European Union^45^ and needing PKP after failure of primary topical treatment. We assumed that BSCVA was age-appropriate before AK. No differences were observed for the parameters of sex, side of the eye and use of CL. However, the patients with poor outcomes were significantly older at first presentation than the patients without poor outcomes. This result is in accordance with previous studies, suggesting an increased prevalence of dry eye in elderly individuals and therefore an increased risk for AK; moreover, host defence in elderly individuals might possibly be altered^29,46^. Poor outcomes were also significantly more frequent in patients with previous treatment with corticosteroids than in those without. Again, these results are in accordance with previous results^29,47^. Previous studies have suggested that the use of corticosteroids is associated with an increased likelihood of treatment failure^29,47^; however, we do not fully agree with this finding. The fact that severe cases may have been treated with corticosteroids needs to be taken in consideration. Whether corticosteroid use led to the increased risk for a poor outcome or whether the cases treated with corticosteroids were more severe than those treated with other medications, even though the ORs might indicate this hypothesis, are impossible to say. Corticosteroids can possibly be initiated after the diagnosis of AK is confirmed and AAT has been administered for at least two weeks^46^. Another interesting finding was that cysts were associated with a decreased risk for a poor outcome. This might be explained by the fact that a cyst is the metabolically inactive and dormant form of Acanthamoeba. Moreover, patients with poor outcomes had longer durations of symptoms before initial presentation, worse BSCVA at initial presentation, and longer follow-up times than those with good outcomes; however, no difference in the number of appointments at our hospital was found.

AK remains a challenge to diagnose and treat. Treatment can be corneotoxic and is often delayed, and pathogens sometimes resist conventional treatment methods. Every patient with a positive history of CL wear, pain and stromal pathology or ring infiltrate should therefore be tested for Acanthamoeba.

The strength of this study is the long observation period and the relatively large sample size. We could follow up clinical courses of 44 eyes with acanthamoeba keratitis over up to 19 years of observation, allowing for good insight into the courses of treatment and complications. The majority of cases showed relatively good results as the necessity for PKP was rather low and restoration of BSCVA was satisfactory in most cases, and only five eyes remained severely visually impaired. Additionally, that patients with AK within our service area were not enrolled in this retrospective study but rather treated by external registered ophthalmologists exclusively is unlikely. Clinical courses of AK in our region can therefore be sufficiently followed, and the development of complications is clearly understood. Results from long-term, retrospective studies like ours are of great importance for rare diseases like Acanthamoeba keratitis, and this study is one of only a few, focusing on an area located in continental Europe. However, some limitations need to be addressed. Patients presented with different stages of disease, and the exact duration of pre-treatment was not always known. Due to the retrospective study design, data was not always completely available.

We highlight the need for awareness of AK among not only ophthalmologists but also the general population for early diagnosis and treatment to maintain good BSCVA and prevent complications and poor outcomes.

## Methods

This retrospective study was approved by the Ethics Committee of the Medical University of Graz, Austria (EC Approval Number: 28-419 ex 15/16). The need for informed consent in adults and parents or guardians from underaged patients was waived by the Review Board of the Ethics Committee. Patient records were anonymised and de-identified prior to analysis. The study was conducted in accordance with the principles and regulations of the Declaration of Helsinki.

A retrospective study design was chosen to include the majority of cases in our region within the last 20 years. The Department of Ophthalmology at the Medical University of Graz is the main ophthalmic tertiary care centre in a region with more than 1.2 million people. As the main ophthalmic tertiary care centre, exceedingly few patients present themselves (only in cases of severe pain or serious injury) to our department; alternatively, patients are referred to us by registered ophthalmologists, or they are referred to us by other departments for the purpose of diagnostics, therapy and surgery. Data were collected from medical and laboratory records in a computer database ranging from 1997, the beginning of electronic documentation, to 2018. We further requested patient records from registered ophthalmologists to complete the remaining course data, obtaining a response rate of 15/24 (62.5%). Cases were only included if laboratory testing was positive for either Acanthamoeba trophozoites or cysts. Diagnostic modalities included corneal swabs, polymerase chain reaction (PCR) assays, anterior chamber (AC) punctures and biopsies. Additionally, smears were performed from CLs and CL containers at follow-up consultations, as earlier studies have indicated them to be a source of Acanthamoeba^48,49^. In many cases, testing for acanthamoeba was repeated, and different testing modalities were used contemporaneously. During the course of the study, a standardized testing regimen for Acanthamoeba, including smears, PCR and culture, was introduced. Data from this regimen were only recently available. In five cases (5/44, 11,4%) no AK testing was conducted at our department, as the patients were referred for the purpose of therapy adjustment and already had positive smears and PCR results.

Acquisition of data was standardized for all patients. The collected data included the dates of the first and final presentations; number of consultations; referral time; follow-up time; age at first consultation; best spectacle-corrected visual acuity (BSCVA) at first consultation, after 1 month, 3 months, 6 months, 1 year, 2 years, 3 years, and 4 years and at the last consultation; intraocular pressure (IOP); status of CL wear and type of CL; previous trauma; confirmation of diagnosis and diagnostic modalities; presence of Acanthamoeba trophozoites and cysts; microbial coinfections (bacteria, fungi, HSV); morphological signs (hypopyon, stromal or ring infiltrates, or anterior synechia,) and symptoms (photophobia, blepharospasm, pain, foreign body sensation, or epiphora); medical therapies; and surgical procedures.

Topical medical therapy in our study population comprised antiamoebic therapy (AAT) and concomitant treatment. Topical AAT included biguanides (PHMB Lavanid 0.02% and chlorhexidine 0.02%) and diamidines (propamidine isethionate Brolene or Golden Eye 0.1% and desomedine 0.1%) with either monotherapeutic or combined use. Within each of the treatment strategies, additional concomitant ointments with potentially antiamoebic or supportive properties were recorded. These included topical steroids, topical antibacterial ointments, topical antiviral ointments, topical antimycotic ointments and further disinfecting ointments (Betaisodona 1:32). The different treatment strategies were defined as biguanide monotherapy, diamidine monotherapy or a combination of both.

Advanced Acanthamoeba infection was defined as signs of ring infiltrates, stromal infiltrates, and hypopyon^12^. Poor outcomes after primary AK therapy were defined as BSCVA equal to or higher than logMAR 0.4 at the final recorded follow-up and/or the need for PKP.

Documented surgical interventions involved PKP and re-keratoplasty as well as amniotic membrane transplantation. Secondary complications consisted of secondary glaucoma and complicated cataracts.

### Diagnostic methods

To achieve a diagnosis of AK, corneal scrapings, AC punctures and biopsies were tested through smears and PCR. Additionally, surface testing of CL and CL containers was performed. Smears were air dried and stained on a specimen slide with Lactophenol Cotton Blue (LPCB) according to the formulation of Thomas and Kuriakose^50^ at our in-house laboratory. Differentiation between trophozoites and cysts was only conducted for distinct findings. Additionally, samples were sent to the Molecular Parasitology, Institute of Specific Prophylaxis and Tropical Medicine, Centre for Pathophysiology, Infectiology and Immunology at the Medical University of Vienna for Acanthamoeba-specific PCR. PCR was performed using the protocol by Schroeder et al*.*^51^. A genotype T4 *Acanthamoeba castellanii* strain was used as a positive control (strain 1BU, ATCC PRA-105), and water lacking DNA was used as a negative control. PCR testing was performed by and according to Walochnik et al*.*^52^.

AK is often a polymicrobial disease. Laboratory testing therefore included testing for bacteria and fungi using Gram staining and PAS staining on cultures grown in blood agar, chocolate agar, Brain–Heart-Infusion Broth, Sabouraud-dextrose agar and non-nutrient agar plates covered with *Escherichia coli*.

### Statistical analysis

For descriptive statistics, continuous parameters are presented as means ± standard deviations (SDs), medians, minimums and maximums. For categorical parameters, frequencies and relative frequencies are used. To investigate the associations of age (categorized into two groups based on median age of 27), sex, presence of cysts and advanced AK signs with time from symptom onset to the initial visit, Mann–Whitney U tests were performed. To compare the parameters between patients who underwent or did not undergo PKP, Mann–Whitney U tests, t-tests and Fisher’s exact tests were used. Visual acuity from the first presentation to the last documented follow-up was compared with paired t-tests. A univariable logistic regression analysis was performed to explore risk factors for poor outcome. ORs and their corresponding 95% confidence intervals (CIs) are presented. Due to the small sample size, dependencies in the data (n = 2 bilateral cases) were not considered in the statistical tests. However, to analyse sensitivity, all tests were repeated randomly, excluding one eye among the bilateral AK cases. The observed results were similar (data not shown). A *p* value of < 0.05 was considered to indicate statistical significance. SAS version 9.4 (SAS institute, Cary, North Carolina, USA) was used for the statistical analysis.

## Data Availability

The datasets and medical records generated during and/or analysed during the current study are available from the corresponding author upon reasonable request. Medical records are not publicly available.
